# An Influence of Immunomodulation on Th1 and Th2 Immune Response in Endometriosis in an Animal Model

**DOI:** 10.1155/2013/849492

**Published:** 2013-11-05

**Authors:** K. Szymanowski, J. Niepsuj-Biniaś, A. Dera-Szymanowska, M. Wołuń-Cholewa, A. Yantczenko, E. Florek, T. Opala, M. Murawski, K. Wiktorowicz

**Affiliations:** ^1^Department of Mother's and Child's Health, K. Marcinkowski University of Medical Sciences, Polna Street 33, 60-535 Poznań, Poland; ^2^Department of Perinatology and Gynecology, K. Marcinkowski University of Medical Sciences, Polna Street 33, 60-535 Poznań, Poland; ^3^Department of Cell Biology, K. Marcinkowski University of Medical Sciences, Rokietnicka 5D Street, 60-806 Poznań, Poland; ^4^The Scientific Research Enterprise RESAN, Street Tolstogo 1, 210026 Vitebsk, Belarus; ^5^Department of Toxicology, Laboratory of Environmental Studies, K. Marcinkowski University of Medical Sciences, Dojazd 30 Street, 60-631 Poznań, Poland; ^6^Department of Obstetrics and Gynecology, Medical University of Wroclaw, Ul. M. Curie-Skłodowskiej 58, 50-369 Wroclaw, Poland; ^7^Department of Biology and Preservation of Environment, K. Marcinkowski University of Medical Sciences, Ul. Długa 1/2, 61-848 Poznań, Poland

## Abstract

*Aim.* To assess the role of the Th1 and Th2 cellular response in the etiology of endometriosis observed in a rat model, with the use of the RESAN immunomodulator. *Materials and Methods.* A comparative analysis of cytokines in blood serum typical of Th1 (TNF-**α** and INF-**γ**) and Th2 (IL-4, IL-6, IL-10) cell response in groups of rats, in which RESAN preparation was used as prophylaxis (Gr. I) or treatment (Gr. II) of endometriosis. *Results.* The results indicated an increase in the level of cytokines in blood serum typical of Th2 cell response by comparing the second and third stages of the experiment in the second group of rats and a decrease in IL-4 and IL-10 between III and IV stages. There was a significant difference in cytokine levels during the third stage of the experiment by comparing I and II groups of rats. In the III group of rats, levels of IL-10 significantly increased between the II and III stages of the experiment. *Conclusion.* RESAN preparation shows Th2 cell response, inhibiting the development of endometriosis in a rat model. Due to successful prophylactic action, one may speculate that RESAN vaccine may be effective as a complementary treatment after surgical excision.

## 1. Introduction

Endometriosis is defined as a chronic, inflammatory, and estrogen-dependent disease, involving the presence of functioning endometrial glands and stroma outside the uterus. It is a benign disease, but due to the accompanying symptoms and chronic nature, it is a very essential medical, social, and economic problem. According to various statistics, the prevalence of endometriosis is 7–17% among reproductive age women [1–4].

One of the theories regarding the cause of endometriosis includes immune alterations that fail to identify and destroy tissue that grows outside of the uterus [5]. Numerous data indicate functional changes in peripheral blood lymphocytes of patients with endometriosis in response to autologous endometrial antigens. It is also known that the cytokines in peritoneal fluid of women with endometriosis stimulate the proliferation of autologous and heterologous endometriosis. On the basis of the presence and activity of selected cytokines typical for various types of cellular response, it was indicated that the development of endometriosis is favored by Th2 cellular response and the associated cytokines, IL-4, IL-5, IL-6, IL-10 among others [6, 7].

The available pharmaceutical treatment does not provide complete and lasting effect in treatment of endometriosis, and the associated side effects have a significant effect on worsening the quality of life of the treated patients. For this reason, there is constant search for new forms of treatment of endometriosis.

For several years, research continues on the RESAN vaccine composed of several components imitating tissue antigens specific for endometrial stroma, fibroids, and the protein CA-125, CA 15-3 (patent no. 5942). Given the results of proving efficacy of RESAN in preventing and possible treatment of endometriosis [8], the next question is, what kinds of mechanisms are involved in the mentioned action of this preparation? Following numerous reports showing the impact of immune system alterations on the etiopathogenesis of the disease, the need to evaluate the possible action of the vaccine on the mentioned system seemed fairly obvious.

## 2. Materials and Methods

**Figure 1:**
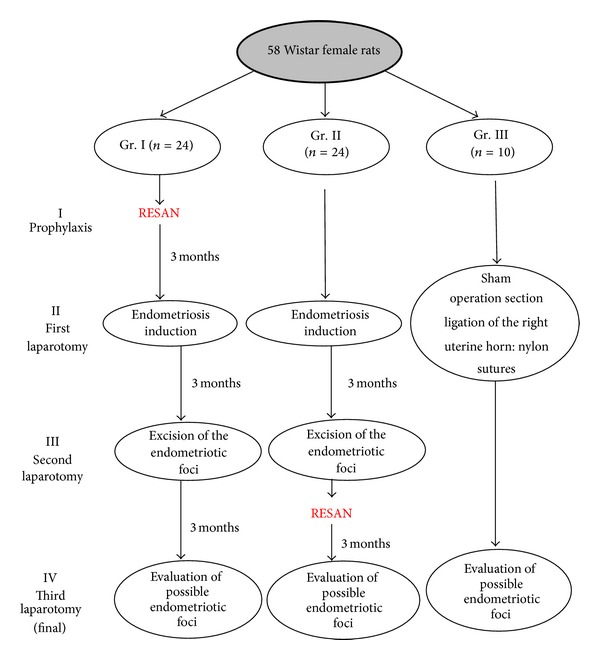
Experimental model of the study.

The research material was serum obtained from 58 adult Wistar female rats during the following four stages of the study (Figure 1).

The study was conducted after approval by the Regional Animal Research Committee. The experiment was performed in accordance with both Ministry and Higher Education Report of 1959 and the UNESCO Declaration of Animal Rights from the 1978 (Paris) guidelines.

Animals were kept in plastic cages on the premises of the Department of Toxicology, University of Medical Sciences, at a constant temperature and humidity; they were fed a constant feeding composition—containing 24% protein. Animals had continuous access to food and drinking water. During subsequent stages of the experiment, blood was collected from the animals' tails in an amount of 2 mL and centrifuged, and the resulting serum was stored at approximately −70°C.

During the first (I) phase of the study (prophylaxis), a group of rats (*n* = 24) underwent vaccination with RESAN (200 mg dose of antigen supplemented with saline to a volume of 1 mL)—half of the dose was given subcutaneously and half intramuscularly into the upper part of the buttock, according to the manufacturer's instructions.

The RESAN vaccine is a complex of molecules extracted from xenogeneic tissues, that is, *G. domesticus*. It contains glycoproteins (fraction a2P), peptides, and carbohydrate fragments imitating more than 40 different tumor antigens (patent no. 5942, [8]).

Following, stage II took place 3 months after endometriosis induction was performed in 48 rats (24 had been vaccinated previously with RESAN, group I, and 24 had not; group II, the treatment group). The process took place in a clean but not completely sterile environment, using microsurgical methods (Heinz operating glasses among others). A mid-ventral laparotomy was performed aseptically under pentobarbital anesthesia. After visualization of the right uterine horn, a 3 cm segment of the uterine horn was ligated and excised. The uterus was closed by hemostatic sutures. After immersing the excised horn in the sterile culture medium, the endometrium was detached from the muscular layer. The obtained graft measuring 4 mm × 4 mm was then attached to the parietal peritoneum on the right side of the abdominal wall using 6–0 nylon sutures.

The abdominal wall was closed with a 1-0 Vicryl running sutures. As in the previous stage, prior to surgery, 2 mL of blood was collected from all rats, centrifuged, and frozen for further proceedings.

In the remaining group of 10 rats (group III, control), a sham operation was performed. After opening the abdominal wall, section and ligation of the right uterine horn was performed. Next, a 6–0 nylon suture was attached to the same place in the abdominal wall as in the study animals. All the procedures were performed in the exactly the same manner as in the group of rats with transplanted endometrium. The blood was collected analogically from the tail, immediately after anesthesia, before surgery.

Three months later, stage III of the study was performed. In all groups relaparotomy was performed, during which possible endometrial foci were identified, describing them in detail, including photographic documentation. Then, excision of the endometriotic foci was performed in groups I and II. Finally, the left uterine horn was excised for parallel investigation. The abdominal wall was closed with 1-0 (Dexon) suture. During all stages of the study, microsurgical techniques were applied. Following, 23 rats from group II were vaccinated with RESAN in the same manner as described in group I. Blood was collected from all animals.

After three months, during the IV stage of the study, third laparotomy was performed in all animals, during which once again the possible endometriosis foci and adhesions were collected for histological evaluation. Blood was collected from all animals. During this stage, in group I we evaluated only the animals in which we have previously diagnosed features of endometriosis (*n* = 5).

Two animals failed to complete the entire experiment: one because of postoperative bleeding two days after the second operation (group I) and the second due to dehiscence and wound infection one day after the first operation (group II).

All collected fragments of endometrial implants were fixed, cut on microtome and stained with hematoxylin, and eosin. Histological assessment by light microscopy was performed by the same person in a “blind manner”; that is, the evaluator did not know the result of macroscopic evaluation and did not know which group of rats is derived from. The study looked for the presence of endometrial glands and endometrial stroma components with hemosiderin loaded macrophages. A second assessment was performed by the consulting histologist (Figures 3, 4, and 5).

The criteria for the diagnosis of endometriosis by the macroscopic evaluation included the presence of one or more of the following characteristics: the presence of cysts filled with clear fluid and/or the presence of thin adhesion around the implant and/or clearly visible hypervascularisation in the form of foci on the abdominal peritoneum.

Uterine fragments obtained during the different stages of experiment were secured for other parallel branches of the experiment.

The serum obtained during the subsequent stages of experiment was checked for the concentration of IL-4, IL-6, IL-10, TNF-*α*, and IFN-*γ* by flow cytometry. The study also evaluated the concentration of rat procalcitonin (PCT) by ELISA.

For statistical evaluation, we have used nonparametric Mann-Whitney test, Kruskal-Wallis test with Dunn's multiple comparison test, Wilcoxon test, and Friedman test with Dunn's multiple comparisons tests, assuming the *P* = 0.05 value to be significant.

## 3. Aim of the Study

The aim of the study is to assess the role of the Th1 and Th2 cellular response in the etiology of endometriosis observed in a rat model, with the use of the RESAN immunomodulator.

## 4. Results

**Figure 2:**
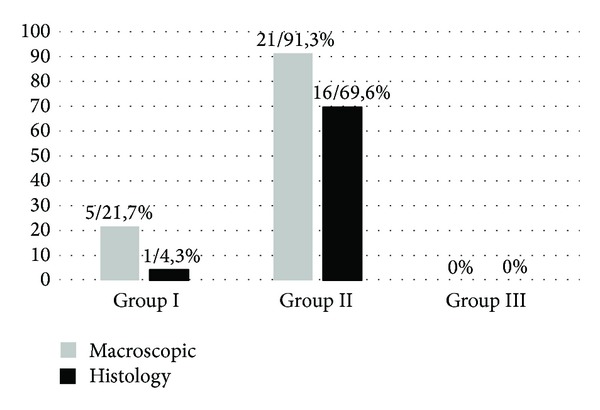
Comparison of positive endometriosis induction (second laparotomy). The bars show a percentage of animals in which endometriosis was diagnosed either macroscopically or by histology. Macroscopic assessment revealed endometriosis in 21.7% and 91.3% (*P* < 0.0001) of the animals in groups I and II, respectively. A positive, histologically confirmed endometriosis was found in 4.3% of the animals in group I and 69.6% in group II (*P* < 0.0001).

**Figure 3:**
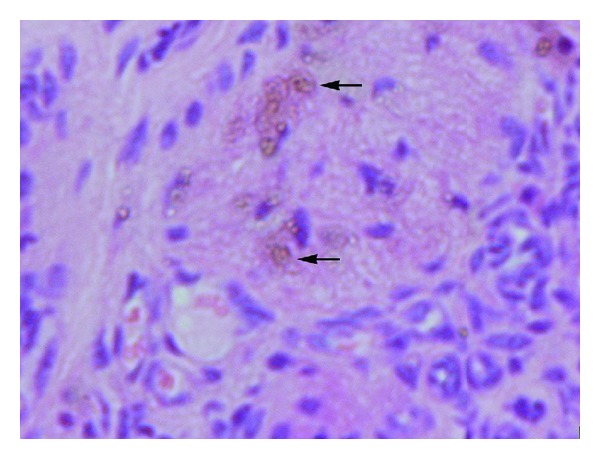
Rats haemosiderin deposits (gr. II).

**Figure 4:**
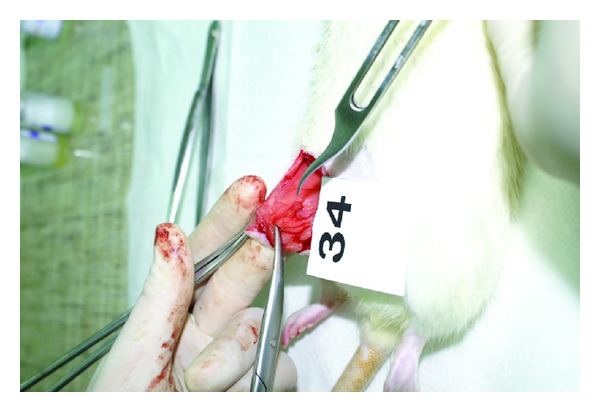
Rats endometriotic lesion on peritoneum (gr. II, st. III).

**Figure 5:**
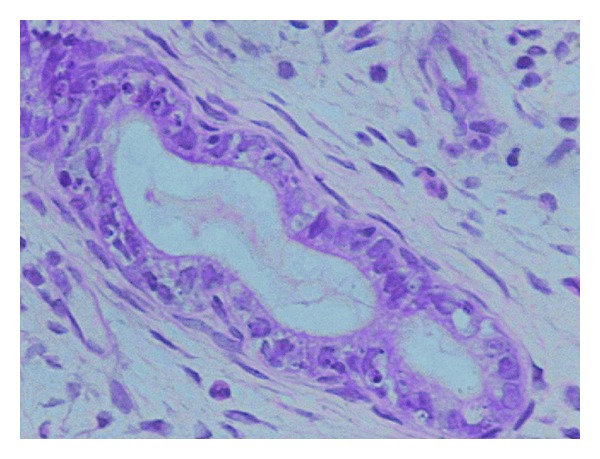
Rats endometrial gland in endometriotic lesion; H E, 400x (gr. II, st. III).

During the third stage of the study, positive histologically confirmed endometriosis was found in 4.3% of the animals in groups I and 69.6% in group II (*P* < 0.0001). Macroscopic assessment revealed endometriosis in 21.7% and 91.3% (*P* < 0.0001) of the animals in groups I and II, respectively. These data are shown in Figure 2. During the fourth stage of the experiment, no signs of endometriosis were found in studied groups on both macroscopic and histological assessments.

**Figure 6:**
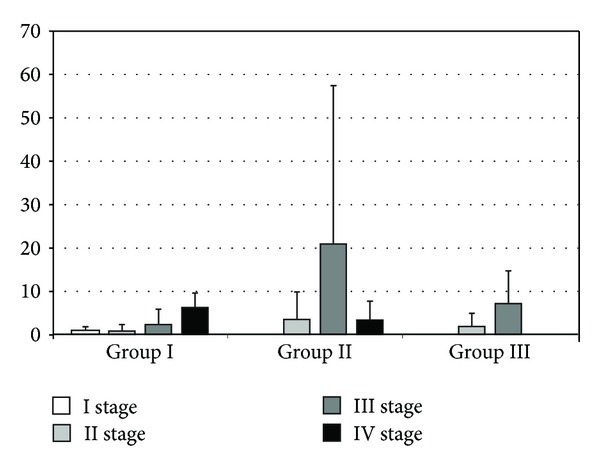
Levels of IL-4 in rat serum (pg/mL). The bars show mean ± SD. In group II, there was a significant increase in IL-4 between stages II and III of the experiment (3.39 ± 6.38 versus 20.81 ± 36.50, *P* < 0.01) and a statistically significant decrease in IL-4 between stages III and IV (20.81 ± 36.5 versus 3.27 ± 4.37, *P* < 0.01). Furthermore, a statistically significant difference in the levels of IL-4 was detected in stage III of the experiment between groups I and II (2.22 ± 3.60 versus 20.81 ± 36.50; *P* = 0.0004).

**Figure 7:**
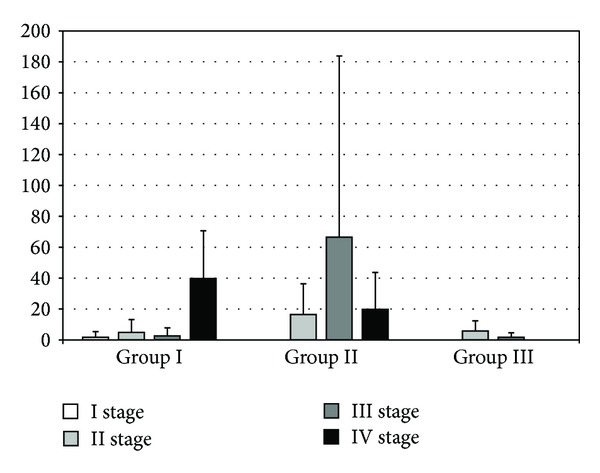
The levels of IL-6 in rat serum (pg/mL). The bars show mean ± SD. A difference in the levels of IL-6 between groups I and II during the stage III of the experiment was observed (2.57 ± 5.27 versus 66.49 ± 117.32; *P* = 0.01).

Comparing the levels of IL-4 in group II, there was a significant increase in cytokines during the third stage compared with stage II (3.39 ± 6.38 versus 20.81 ± 36.50, *P* < 0.01) and statistically significant decrease in IL-4 by comparing the third and fourth stages in group II (20.81 ± 36.5 versus 3.27 ± 4.37, *P* < 0.01). During the third part of the experiment, there was a significant difference between IL-4 concentrations in the serum of rats (2.22 ± 3.60 versus 20.81 ± 36.50) in groups I and II, respectively (*P* = 0.0004) (Figure 6). Analyzing the level of IL-6, an increasing trend was observed in the second group of rats by comparing the second and third phase of the experiment (16.40 ± 19.95 versus 66.49 ± 117.32; NS). We have also observed a difference in the level of IL-6 between groups I and II during the third stage of experiment, which was during the diagnostic relaparotomy after implantation of ectopic endometrium (2.57 ± 5.27 versus 66.49 ± 117.32; *P* = 0.01) (Figure 7).

**Figure 8:**
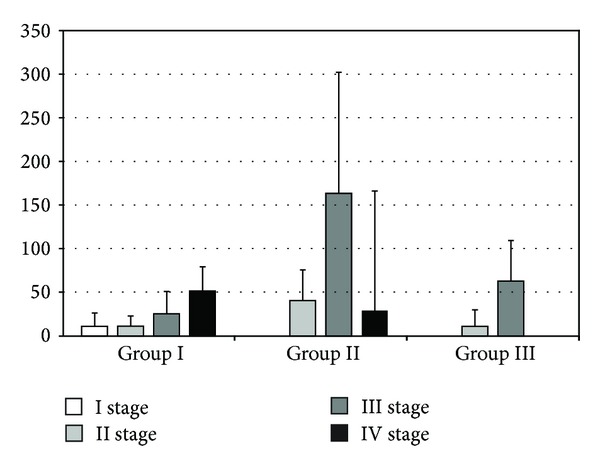
The levels of IL-10 in rat serum. The bars show mean ± SD (pg/mL). A statistical significance was observed in group II between stages II and III (40.58 ± 35.38 versus 163.55 ± 139.06; *P* < 0.01) and between stages III and IV (163.55 ± 139.06 versus 29.09 ± 33.06; *P* < 0.01) of the experiment. Furthermore, a significant increase was observed in group III between stages II and III of the experiment (11.12 ± 18.93 versus 62.99 ± 46.53; *P* = 0.01). A statistically significant difference was observed in stage II of the experiment between groups I and II (11.27 ± 11.84 versus 40.58 ± 35.38; *P* = 0.02) and between groups II and III (40.58 ± 35.38 versus 11.12 ± 18.93; *P* = 0.01). In the third stage of the experiment, a difference between groups I and II was observed (25.37 ± 25.889 versus 163.55 ± 139.06; *P* = 0.00002).

**Figure 9:**
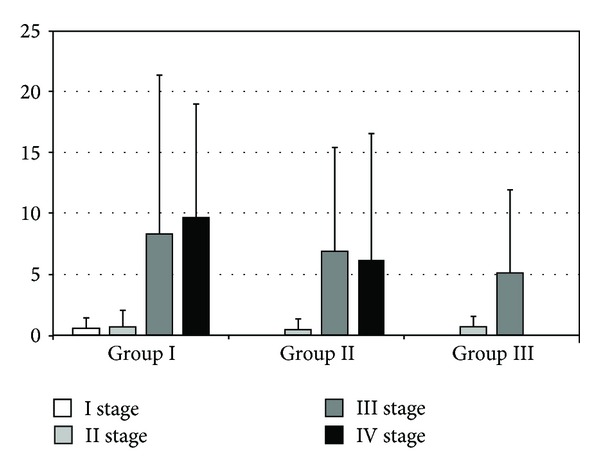
The levels of IFN-*γ* in rat serum. The bars show mean ± SD (pg/mL). A statistically significant increase in the concentration of cytokines was observed in group II between stages II and III of the experiment (0.44 ± 0.90 versus 6.87 ± 8.55; *P* < 0.01) and stages II and IV (0.44 ± 0.90 versus 6.16 ± 10.38; *P* < 0.01). Other visible differences did not reach statistical significance.

There were significant differences in levels of IL-10 during different parts of the experiment. In group II, we have observed a statistically significant difference between II and III (40.58 ± 35.38 versus 163.55 ± 139.06; *P* < 0.01) and III and IV (163.55 ± 139.06 versus 29.09 ± 33.06; *P* < 0.01) stages of experiment. Comparing the second and third stage of the study in the third group of rats, we have also observed a statistically significant increase in IL-10 (11.12 ± 18.93 versus 62.99 ± 46.53; *P* = 0.01). Comparing the cytokine levels between groups during the second phase of the experiment, there was a significant difference between groups I and II (11.27 ± 11.84 versus 40.58 ± 35.38; *P* = 0.02) and between II and III (40.58 ± 35.38 versus 11.12 ± 18.93; *P* = 0.01). The analysis of the level of IL-10 in the third stage of the experiment indicated difference between first and second groups of rats (25.37 ± 25.889 versus 163.55 ± 139.06; *P* = 0.00002) (Figure 8). Analyzing the level of IFN-*γ* in both I and III groups of rats, there was no significant statistical difference. However, there was an increase in the concentration of cytokines in the second group of rats between II and III (0.44 ± 0.90 versus 6.87 ± 8.55; *P* < 0.01) and II and IV (0.44 ± 0.90 versus 6.16 ± 10.38; *P* < 0.01) stages of the experiment. When comparing the levels of IFN-*γ* between the two groups during the various stages of the study there were no statistically significant differences (Figure 9).

Comparing the levels of TNF-*α* in different groups and at different stages of the experiment, there were no statistical differences. Cytokine levels were below the detection level or reached very low values. On the basis of the obtained results we observed that the level of rat procalcitonin did not vary significantly either within the groups of rat, or depending on the phase of the experiment within a group. TNF-*α* and procalcitonin results are not included; however they can be obtained from the author through e-mail.

## 5. Discussion

The aim of this study was to find out what is first—endometriosis or immunological defect? The method used was rat endometriosis with evaluation of concentration and interactions between selected parameters characteristic for different types of immunological response. The research on the development of effective treatment of endometriosis, especially in the immunological aspects, lasted for years—still with unsatisfactory results. This entails making further attempts to develop an effective treatment of this disease. This action requires an appropriate experimental model which will allow proper evaluation of the impact and possible mechanisms of interaction of the investigated preparations and substances. The experimental model of rat endometriosis has been known for many years. Wistar laboratory rat was found to be a convenient pet because of short (4-5-day) and regular cycles, high availability, and relatively low cost of cultivation [9].

Attempts to evaluate the superiority of chosen cytokines in the development of endometriosis have been undertaken repeatedly. Among the known and studied dysfunctions of the immune system in the course of endometriosis, one has observed reduced NK cell activity in serum and peritoneal fluid [2, 10] and increased activity of peripheral blood monocytes and macrophages in peritoneal fluid. This results in increased production of proinflammatory cytokines, both at rest and after stimulation, among which we should mention TNF-*α*, IL-6, and IL-8 or prostaglandins PGF, PGE [10]. Based on observations of the types of cytokines released in the course of endometriosis, it was shown that there is a relative increase in cytokines characteristic of Th2 cell response and impaired Th1 cell response [6, 11].

Among the evaluated parameters, in our experiment we found cytokines typical for Th1 cell response, that is, IFN-*γ* and TNF-*α*, and cytokines characteristic for Th2-type responses, such as IL-4, IL-6, and IL-10. It turned out that the group of rats not treated with immunoprophylaxis by RESAN preparation showed clearly stronger tendency to elevated levels of cytokines typical for Th2 cell response. Furthermore, the levels decreased after surgery combined with the therapeutic use of RESAN. This would suggest the positive effects of the treatment applied on the immunomodulatory mechanisms important in the battle against endometriosis. Surprisingly, we found no such relation in terms of effects on cytokines typical for Th1-type responses and thus TNF-*α* and IFN-*γ*.

According to the literature, elevated levels of TNF-*α* in peritoneal fluid of patients with endometriosis stimulate the adhesion and implantation of ectopic endometrial cells to the peritoneal walls [7, 12]. The results obtained by us did not indicate different levels of TNF-*α* within each group and at each stage of the experiment. Unfortunately, the vast majority of tests indicated that cytokine levels were below the detection level. Podgaec et al. described analogous results in his study, also finding no statistical difference between the level of this cytokine in the peritoneal fluid and serum among healthy women and women with endometriosis [11]. IFN-*γ* is an important factor which stimulates the adhesion of ectopic endometrial cells to the peritoneum through the stimulation of adhesion molecule ICAM-1 (intercellular adhesion molecule-1) [13]. Nishida et al. described IFN-*γ* as a factor which reduces the tendency of ectopic endometrial cells to apoptosis, thereby enhancing the ability to experience pathological cells outside the uterine cavity [14]. The results obtained by us did not show statistically significant differences between the concentrations of this cytokine within the groups studied. The most important conclusion drawn by the above results may be that immunomodulation acquired with RESAN blocked enhancement of TNF-*α* and IFN-*γ* production, thus stopping nearly completely early endometriosis development. It is well known that these early steps are crucial in the entire process.

Interleukin-6 (IL-6) is an important regulator of inflammation and immune responses. It is released locally by ectopic endometrial implants as well as newly developed endometrial foci and contributes to neovascularization of the implants of endometriosis. In turn, it stimulates the peritoneal fluid T and B lymphocytes to produce autoantibodies in endometriosis, exacerbating the pathological effects of this condition such as infertility [15].

Significantly lower concentration of IL-6 in the first group of rats after implantation of ectopic endometrium can be explained in two ways. In this group only in one case we have confirmed endometriosis, and IL-6 may be produced locally within foci of endometriosis. On the other hand, probably under the influence of immunomodulating actions of RESAN preparation, there was no pathological, excessive activation of macrophages, which could result in increased production of IL-6 by these cells.

Interleukin-4 is released locally and stimulates proliferation of endometriotic tissue, contributing to the formation of adhesions in the course of endometriosis. Moreover, it has a synergistic effect with TNF-*α* in the induction of mentioned proliferation [16]. These observations demonstrate the important role of IL-4 in the development of endometriosis. The results of our experiment can confirm this observation. Once again the question remains what is first? One may conclude that immunomodulation blocked self-promoting mechanisms in early endometriosis development causing huge difference in endometriosis induction between groups. Confirmation of this phenomenon may be the decrease of the IL-4 after endometriosis excision combined with immunomodulation.

Our results on IL-10 demonstrate immunomodulatory effects of RESAN preparation on decreasing the cytokine function and thus Th2 cell response. According to Wu and Ho, IL-10 is produced in increased amounts by activated macrophages in peritoneal fluid of patients with endometriosis [13]. It serves an important regulatory function, by inhibiting the release of cytokines typical for Th1 lymphocytes.

Preliminary results on the effect of RESAN preparation on endometriosis in a rat model appear to be encouraging and certainly mobilize to make further observations. The results regarding the assessment of chosen cytokine concentrations indicate the immunomodulatory effect of this preparation but in the absence of evidence proving the impact of RESAN preparation on TNF-*α* level, and thus one of the main factors responsible for the implantation of endometriosis is also likely that other mechanisms have an effect on the etiopathogenesis of this disease.

What seems to be the most important results of our studies? For years unanswered question: “what is first?” Immunological status plays a crucial role in endometriosis, thus enabling adhesion and survival of displaced endometrial fragments. Moreover, immunomodulation is effective in endometriosis prophylaxis and may be effective as a complementary treatment after surgical excision.
